# Creating Disease Resistant Chickens: A Viable Solution to Avian Influenza?

**DOI:** 10.3390/v10100561

**Published:** 2018-10-15

**Authors:** Fong Yang Looi, Michelle L. Baker, Thomas Townson, Mathilde Richard, Ben Novak, Tim J. Doran, Kirsty R. Short

**Affiliations:** 1 School of Chemistry and Molecular Biosciences, The University of Queensland, St Lucia, QLD 4072, Australia; f.looi@uq.net.au (F.Y.L.); thomas.townson@uq.net.au (T.T.); 2 CSIRO, Australian Animal Health Laboratory, Health and Biosecurity Business Unit, Geelong, VIC 3219, Australia; michelle.baker@csiro.au (M.L.B.); ben.novak@csiro.au (B.N.); timothy.doran@csiro.au (T.J.D.); 3 Department of Viroscience, Erasmus Medical Centre, 3015 GD Rotterdam, The Netherlands; m.richard@erasmusmc.nl; 4 Australian Infectious Diseases Research Centre, The University of Queensland, St Lucia, QLD 4072, Australia

**Keywords:** avian influenza, chickens, transgenics, GMO, poultry

## Abstract

Influenza A virus (IAV) represents an ongoing threat to human and animal health worldwide. The generation of IAV-resistant chickens through genetic modification and/or selective breeding may help prevent viral spread. The feasibility of creating genetically modified birds has already been demonstrated with the insertion of transgenes that target IAV into the genomes of chickens. This approach has been met with some success in minimising the spread of IAV but has limitations in terms of its ability to prevent the emergence of disease. An alternate approach is the use of genetic engineering to improve host resistance by targeting the antiviral immune responses of poultry to IAV. Harnessing such resistance mechanisms in a “genetic restoration” approach may hold the greatest promise yet for generating disease resistant chickens. Continuing to identify genes associated with natural resistance in poultry provides the opportunity to identify new targets for genetic modification and/or selective breeding. However, as with any new technology, economic, societal, and legislative barriers will need to be overcome before we are likely to see commercialisation of genetically modified birds.

## 1. Introduction

In 2016, amid much controversy, the first genetically modified animal for human consumption was approved for sale [1]. The availability of AquAdvantage salmon (engineered to grow much faster than non-genetically modified salmon) to Canadian consumers has ignited numerous debates about the ethics, safety, and feasibility of genetically modified animals [1]. However, this landmark decision has also paved the way to potentially bring other genetically modified animals to market. Possible examples include mastitis-resistant livestock [2] and pigs resistant to African swine fever [3]. Given that in the USA alone, 54 kg of poultry are consumed per capita per year [4], it is of little surprise that there has also been significant interest in using genetic engineering to create disease resistant poultry. Here, we discuss the feasibility of creating genetically modified chickens resistant to avian influenza. We highlight potential genes of interest to confer viral resistance as well as the issues that may limit their widespread implementation. Finally, we explore the potential role that selective breeding may play in the creation of disease resistant poultry.

## 2. Avian Influenza Viruses

Influenza A virus (IAV) represents a significant threat to poultry populations worldwide. A recent outbreak of IAV in South Korea has forced the euthanasia of more than 10% of the country’s domestic poultry population [5]. Similarly, 2014–2015 outbreaks of IAV in the United States caused economic losses as high as $3.3 billion [6]. These included the costs associated with production losses, implementing appropriate disease prevention measures and the indirect costs associated with a loss of confidence in the sector [7]. IAVs are classified into different subtypes based on the antigenicity of the two surface glycoproteins: the haemagglutinin (HA) and the neuraminidase (NA). A total of 16 HA (H1–H16) and nine NA (N1–N9) subtypes have been identified in the natural reservoirs of IAV, wild waterfowl. From here, IAV can spread to gallinaceous poultry. In gallinaceous poultry IAV typically causes a subclinical/mild infection and is referred to as low pathogenic avian influenza (LPAI). However, LPAI H5 and H7 viruses can evolve in gallinaceous poultry to become highly pathogenic avian influenza (HPAI) viruses. This evolution happens via the insertion of a multi-basic cleavage site in the viral HA. Proteolysis is required to cleave the precursor of the viral HA, the HA0, into the functionally active H1 and H2 subunits. Cleavage can be achieved by one of two types of proteases: trypsin-like proteases (limited to the gut and respiratory tract) and subtilisin-like proteases (present in numerous different organs). The presence of a multi-basic cleavage site in HPAI viruses means that these viruses can be cleaved by ubiquitously expressed subtilisin-like proteases. HPAI viruses, thus, cause a fatal and systemic infection in gallinaceous poultry that is characterised by endothelial cell infection, oedema and haemorrhaging [8]. Following their emergence in chickens, HPAI viruses can spread to other avian or non-avian hosts (including humans). HPAI viruses can also further diversify via the process of reassortment [9]. Since 2013, this process of reassortment has led to the diversification of the HPAI H5N1 virus clade 2.3.4.4 [10]. This clade has undergone reassortment with various LPAI viruses to give rise the H5Nx viruses—so called because of the variety of different NA subtypes that have reassorted with the H5 HA. This process has facilitated the emergence of novel HPAI viruses, such as H5N2, H5N6, and H5N8, all of which have caused significant mortality and economic losses [10].

In light of the dramatic effect that HPAI viruses have on gallinaceous poultry populations worldwide, there has been significant interest in the utility of both synthetic and natural antivirals in poultry. However, the use of antivirals can be expensive on a flock level and facilitate the emergence of resistant mutants (for a full discussion of these issues see [11,12]). To address some of these limitations, poultry vaccination programs have been implemented in numerous different countries. Vaccination can be an effective tool in reducing the spread of HPAI viruses, in particular in the context of a sudden outbreak of influenza. Indeed, Vietnam has dramatically reduced the number of HPAI H5N1 cases in gallinaceous poultry by combining vaccination with an improved biosecurity program [13,14]. However, wide-spread vaccination campaigns have failed to completely eradicate IAVs in gallinaceous poultry, and the successful implementation of vaccination programs is associated with numerous challenges. In some cases, the high labour costs associated with vaccination can be prohibitive. This is particularly true for broiler chickens, where their short life-span disincentives vaccine use [15]. Numerous factors (e.g., co-infection with other pathogens, age, and nutritional status of the bird) can also impact vaccine efficacy and reduce vaccine-induced protection [15]. Much like the seasonal human influenza virus vaccine, poultry IAV vaccines require regular updating due to antigenic changes in the virus over time [15]. The absence of sterilizing immunity also raises concerns about vaccination accelerating the process of antigenic drift [7,16]. Finally, vaccination also has important consequences for poultry export markets, as the export of gallinaceous poultry is often restricted from countries that vaccinate poultry against IAV (due to concerns that vaccination may mask an active infection) [15]. These limitations suggest that whilst vaccination programs can help contain IAVs in poultry, vaccination in and of itself is not sufficient to protect the world’s gallinaceous poultry from influenza.

## 3. Genetically Modified Chickens Resistant to Avian Influenza Viruses

**Figure 1:**
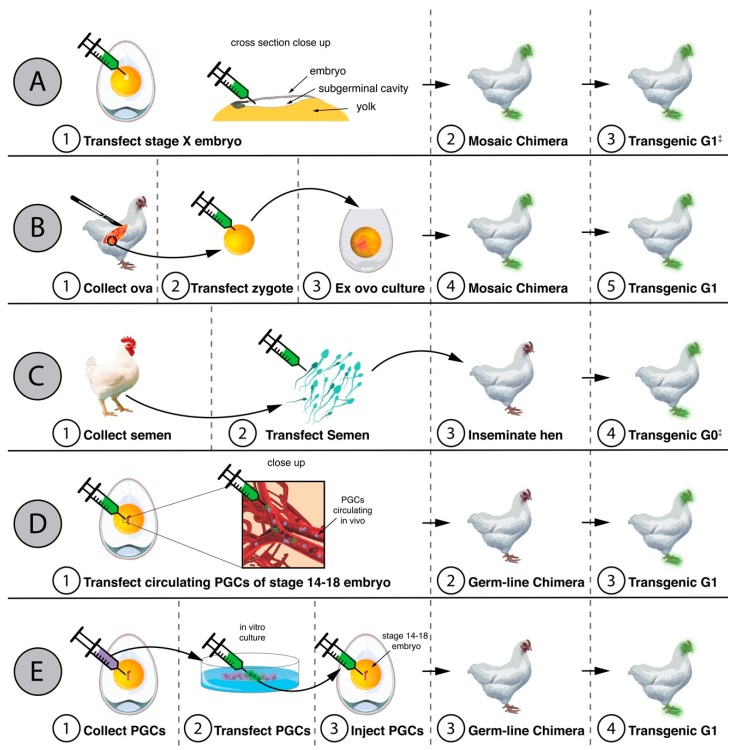
Methods of establishing germ-line modifications in the chicken. (**A**–**E**) show the workflow of integrating a transgene (e.g., GFP reporter gene) into the chicken genome, via transfection with a viral agent to the embryonic disc of a stage X chicken embryo (**A**), microinjection of transfection agents into a single cell zygote (**B**), transfection of sperm for artificial insemination (**C**), transfection of primordial germ-cells (PGCs) in vivo (**D**), and transfection of PGCs in vitro (**E**). Methods C–E have also been successful in generating targeted gene edits in the germ-line of chickens via zinc-finger nucleases, TALENs or CRISPR-Cas9. ‡ denotes methods capable of producing fully transgenic chickens in a single generation.

Given the limitations associated with poultry vaccination, there have been numerous attempts to use genetic modification to protect gallinaceous poultry from IAV. The first such attempt was reported by Lyall and colleagues in 2011 [17]. Using a lentiviral system (see Figure 1), Lyall and colleagues introduced cDNA of an RNA hairpin molecule into the chicken genome [17]. This ‘decoy’ genetic material contained the cDNA of the conserved 3′ and 5′ terminal sequence of the IAV genome [17]. This sequence forms a binding site for the IAV polymerase and its presence inhibited viral polymerase activity in vitro [17]. Importantly, chickens expressing the decoy cDNA were still susceptible to direct infection with the HPAI virus A/turkey/Turkey/1/2005 (with no significant difference observed in the time to death between transgenic and non-transgenic birds) [17]. However, transgenic chickens were less likely to transmit the virus to either transgenic or non-transgenic co-housed birds [17]. These data suggest that this approach, whilst not preventing the emergence of HPAI, may make an infected bird a ‘dead end host’ and significantly reduce the spread of HPAI. It remains to be determined whether the presence of this decoy RNA would prevent the initial transmission of LPAI viruses from wild birds to poultry. This remains a key question for poultry health. Whilst LPAI infections are typically mild, they can increase the risk of secondary bacterial infections [18] and a recent report suggested that at least some LPAI viruses can cause systemic and fatal disease in turkeys [19]. Thus, assessing the protective effects of a transgene on both LPAI and HPAI is essential prior to any attempts at commercialisation. 

The viability of using a transgenic approach to protect against LPAI was subsequently addressed by Byun and colleagues [20]. In these studies, chicken were engineered to express the 3D8 single chain variable Fragment (scFv) customised to have an affinity for the IAV nuceloprotein. scFv was of particular interest as it causes nuclease activity and has displayed hydrolytic activity against both RNA and DNA viruses [20]. Following direct infection with LPAI A/Korean native chicken/Korea/K040110/2010(H9N2), scFv transgenic chickens did not display any difference in oropharyngeal shedding when compared to their non-transgenic counterparts [20]. However, contact-exposed transgenic chickens had significantly lower viral titres in the cloaca and oropharynx compared to non-transgenic birds [20]. Unlike the more targeted approach of Lyall and colleagues [17], the presence of a scFv transgene may serve to protect poultry from a wide variety of different infectious organisms, beyond just IAV [20]. However, scFv levels must be tightly controlled as overexpression can result in the degradation of host nucleic acids [20]. 

## 4. RIG-I: A Transgene of Interest?

The aforementioned studies suggest that genetic modification is a technically feasible approach to reduce the spread of IAV amongst gallinaceous poultry. However, in addition to the insertion of ‘antiviral’ genetic material into the chicken genome, genetic engineering may also be used to improve the immune response of gallinaceous poultry and thus reduce IAV severity. Such an approach is based on numerous comparative immunology studies showing that unlike ducks (who are largely resistant to severe HPAI virus infections), chickens lack or poorly express several key antiviral factors [8,21,22]. Of these antiviral factors, the absence of a chicken retinoic acid-inducible gene (*RIG-I*) has received the greatest attention. RIG-I belongs to a family of DExD/H box RNA helicases and serves to sense viral dsRNA and 5-ppp-ssRNA. In mammalian cells, activation of RIG-I results in the downstream activation of transcription factors, IRF3 and 7 and ultimately the expression of type I interferon, type III interferons and interferon stimulated genes. Comparative genomics suggest that whilst the duck genome has a *RIG-I* homologue with 53% amino acid identity to human *RIG-I*, 78% identity to the zebra finch *RIG-I* and 78.27% identity to the pigeon *RIG-I* [23], there is no chicken homologue of *RIG-I* [22]. This is true of both the Red Jungle Fowl genome and modern breeds of chicken [22], suggesting that chickens lost *RIG-I* and/or its homologues prior to domestication. Indeed, it has been suggested that *RIG-I* loss may have occurred in the stem lineage of Galliformes [24]. Consistent with this genomic analysis, chicken embryonic fibroblasts failed to respond to RIG-I ligands [22]. However, this effect could be reversed by the transfection of chicken cells with the duck RIG-I [22]. Indeed, chicken cells transfected with duck RIG-I upregulated many innate anti-viral genes (including *MX1*, *PKR*, *OASL* and *IFN-β*) and decreased influenza virus replication [22]. Together, these data suggest that a functional *RIG-I* could help protect chickens from severe disease upon infection with IAV. Accordingly, the creation of a transgenic chicken expressing the duck RIG-I has been under patent since 2010 (US20110247091A1). However, it is important to recognise that even in the absence of a *RIG-I* homologue, chickens still mount a pronounced antiviral response to HPAI virus infections in vivo [25,26]. Indeed, it has been suggested that melanoma differentiation factor (MDA5), a RIG-I like sensor, is able to compensate for the absence of *RIG-I* in chickens [27]. Understanding anti-viral signalling in chickens in the absence of *RIG-I* thus remains an important priority for future research. 

## 5. Other Possible Immune Transgenes

In addition to *RIG-I*, there are numerous other immune and non-immune genes that could potentially be expressed/overexpressed in chickens to improve resistance to HPAI viruses. These include, but are not limited to, cyclophilin A [28], interferon stimulated gene 15 [29], viperin [30], heat shock cognate protein 70 [31], and Erb3-binding protein [32]. Interestingly, the utility of overexpressing interferon-stimulated gene 58 (*ISG58*) in poultry has recently been assessed [33]. Rohaim and colleagues [33] used an avian sarcoma-leukosis virus (RCAS)-based gene transfer system to constitutively express chicken *ISG58* (which, in wild-type chickens, is only interferon inducible). Unlike genome edited-based transgenics (e.g., TALEN or CRISPR) RCAS-edited gene delivery resulted in predominant transgene expression in epithelial enriched organs [33]. Nevertheless, *ISG58* transgenic chickens were protected from challenge with 10^4^ egg infectious dose 50 (EID_50_) of HPAI H5N1 A/chicken/Egypt_128s_2012 [33]. Specifically, unlike control chicks, infected RCAS-chIFIT5 transgenic chicks did not succumb to infection, displayed reduced histopathological changes and reduced duration and titres of viral shedding [33]. Moreover, the overexpression of *ISG58* also protected chicks from 10^5^ EID_50_ of velogenic Newcastle Disease Virus (vNDV) [33]. However, overexpression of *ISG58* was, in and of itself, insufficient to protect chicks from a lethal dose of either a HPAI H5N1 virus (10^6^ EID_50_) or vNDV (10^6^ EID_50_) [33]. Moreover, there were significant developmental problems observed in chickens that constitutively expressed *ISG58* [33]. The hatchability of transgenic eggs was compromised compared to wild-type eggs, with nine chicks having to be humanly euthanised prior to reaching 12 days old [33]. Moreover, transgenic chicks had a significant lower hatching weight compared to wild-type chicks [33]. Whether similar limitations will impair the commercialisation of other immune based, transgenic approaches remains to be determined. However, the growing availability of a wide variety of different, high-quality avian genomes will facilitate the identification of additional components of the anti-viral response that are impaired/missing in gallinaceous poultry and could represent attractive targets for genetic modification.

## 6. Limitations of Transgenics

Despite the recent commercialisation of AquaAdavantage Salmon, it must be recognised that it took over 25 years to bring this product to market [1]. This simple fact suggests that whilst disease-resistant poultry are technically possible, that there are still significant economic, environmental, societal and legislative barriers to their commercial implementation. As discussed above, an important factor to consider in this regard is the commercial viability of these animals. Whilst transgenic chickens expressing decoy cDNA had no obvious differences in hatch weight [17], their viability as meat or egg-producing chickens remains to be determined. Similarly, given the high mutation rate of influenza, one must be aware of the potential of resistant mutants arising in response to any transgenic modifications. The risk of this occurring is minimised with strategies that target highly conserved components of the virus (such as the viral polymerase and/or nucleoprotein). However, it still remains possible that over time the widespread use of transgenic chickens could create a strong selective pressure for resistant viral variants. Finally, consumer concerns about genetically modified animals (including questions about their long-term impacts on health and environment) should not be overlooked [34]. Consumers in countries where there is a low risk of avian influenza may feel that the perceived risks of genetic modification outweigh any of the benefits of disease resistance. It is more likely that there will be lower consumer resistance to genetic modifications in countries where avian IAVs are endemic. However, the introduction of genetically modified chickens in any one country will have important knock-on effects in terms of trade and poultry export, all of which must be taken into consideration prior to any commercialisation attempts. In this regard, it may be that a strategy which only seeks to modify *existing* genes such that they can be reverted to an ancestral sequence would be more attractive to both consumers and regulatory boards. This strategy of ‘genetic restoration’ could potentially be of significant benefit in the context of IAV, as it is well established that poultry domestication has resulted in decreased immune function and increased susceptibility to infectious diseases [35]. The technology underpinning genetic restoration is already available (see Figure 1) and this remains an option for the future. 

## 7. Alternative Approaches to Genetic Modification: Selective Breeding

Selective breeding for commercially important traits such as egg numbers, persistency in lay and shell colour is standard practice in the poultry industry [36]. Given the issues surrounding the commercialisation of genetically modified organisms, selective breeding for resistance to IAV may represent an attractive alternative approach to develop disease resistant poultry. Of course, such an approach depends on the presence of individual birds or breeds of poultry that display differential susceptibility to IAV. Whilst this area of research still remains somewhat in its infancy, in outbreaks of HPAI viruses there are often a proportion of birds who survive the outbreak [6,37], suggesting that host-dependent differences can influence disease outcome. Similarly, certain breeds of chickens are known to be inherently less susceptible to IAV [38,39,40]. For example, there was a significant difference in viral shedding between two inbred lines of chicken infected with LPAI A/Turkey/England/647/77(H7N7) [38]. Similarly, Thai indigenous chickens shed significantly less HPAI H5N1 than White Leghorns [39] whilst Fayoumi chickens shed significantly less LPAI H5N2 than Leghorn chickens [40]. The genetic basis for this differential susceptibility remains incompletely understood, although it has been associated with host-dependent differences in innate immunity [38]. Indeed, there have been suggestions that breed-dependent single nucleotide polymorphisms (SNPs) in the *MX1* gene alter susceptibility to IAV. In mammals, MX1 is an interferon-induced GTPase that inhibits IAV transcription and replication. Initially, the chicken MXI was reported to have no antiviral activity [41]. However, there are several natural mutations in the chicken *MX1* [42]. One of these mutations, S631N, is associated with increased antiviral activity [42]. The S631N mutation is at a higher frequency in Chinese native chicken breeds compared to commercial lines [43], suggesting that this antiviral trait may have been lost during chicken domestication. In vivo, the *MXI* Asn631 allele conferred resistance to a HPAI H5N2 virus [44]. However, whether the S631N mutation truly increases antiviral activity remains controversial. In vitro, the N631 allele did not inhibit IAV replication in chicken primary embryo fibroblasts [45]. In ovo, no association was observed between the *MXI* genotype and the pathogenesis of IAV [46]. In vivo, the severity of a HPAI H7N1 virus in chickens was not affected by the *MXI* genotype [47]. These data suggest that IAV resistance is a multifactorial trait, and that any protective effect of the S631N mutation may have to take place in a specific genetic background.

Greater insight as to the genetic basis for natural disease resistance has been gleaned from genome wide association studies (GWAS) comparing birds that survived and succumbed to outbreaks of IAV [6,37]. For example, in White Leghorn chickens, survival during an HPAI H5N2 virus outbreak was associated with SNPs in genes associated with the immune response [6]. These included genes for the TNFα receptor, interferon kappa and the signal peptide peptidase like 2B (associated with regulation of the adaptive immune response) [6]. In contrast, SNPs in the genes for Neuroligin 4 and Neurexin 3 were associated with survival following a HPAI H7N2 virus outbreak [37]. Whilst these data suggest that not one single genetic signature determines survival, a role for neuronally-associated genes in disease severity echoes observations from mammalian studies that the nervous system helps regulate the inflammatory response [48]. Together, these data suggest that studies of natural genetic diversity and the underlying alleles may serve as a complementary approach to genetic modification, either by increasing disease resistance and/or identifying key genes for genetic restoration.

## 8. Conclusions

The recent outbreaks of H5Nx viruses across the world highlight the continual threat that avian influenza viruses pose to both poultry and human health. Rapid advances in genomics and gene editing suggest that disease resistant, genetically modified chickens could represent a viable solution to the problem of avian influenza. However, whilst the creation of influenza resistant chickens is technically possible, there still remain many barriers in place blocking their commercial implementation. These include issues such as consumer concerns and a detailed assessment of their commercial and environmental viability. It is possible some of these issues can be circumvented and/or mitigated with alternative approaches such as selective breeding and/or genetic restoration. However, regardless of the methodology used, novel approaches to address the issue of avian influenza must remain a global health priority.
